# Navigating the Unknown: A Surgical Perspective on Thoracic ALK-Positive Histiocytosis With Literature Review

**DOI:** 10.7759/cureus.117571

**Published:** 2026-10-07

**Authors:** Ramzi Addas, Ahmed Alsabban, Shahad Almarwan, Saud Alharbi

**Affiliations:** 1 Department of Thoracic Surgery, King Abdulaziz Medical City, National Guard Hospital, Jeddah, SAU; 2 Department of General Surgery, King Abdulaziz Medical City, National Guard Hospital, Jeddah, SAU

**Keywords:** alk-positive histiocytosis, aph, extra-pulmonary alk-positive histiocytosis, pulmonary mass, wedge resection

## Abstract

Anaplastic lymphoma kinase (ALK)-positive histiocytosis (APH) is a rare disorder characterized by the accumulation of cells derived from monocyte-macrophage-dendritic cells. Thoracic APH is incredibly unusual and thus creates a diagnostic challenge. In this study, we report a case of extrapulmonary APH that was managed with mass excision, with no further treatment. Moreover, we reviewed previously published relevant adult ALK-positive histiocytosis cases, their primary management, and the disease progression.

## Introduction

Histiocytic neoplasms comprise a heterogeneous group of rare disorders characterized by the clonal proliferation of cells derived from the monocyte-macrophage-dendritic cell lineage. While entities such as Langerhans cell histiocytosis (LCH) and Erdheim-Chester disease (ECD) are well characterized, the classification of histiocytic disorders has evolved significantly with the identification of recurrent molecular alterations, particularly involving the MAPK signalling pathway [1].

More recently, rearrangements involving the anaplastic lymphoma kinase (ALK) gene have led to the recognition of ALK-positive histiocytosis (APH) as a distinct clinicopathological entity. First described in early infancy in 2008, the recognized clinical spectrum has since expanded to include patients of all ages, ranging from localized disease to multisystem involvement - most commonly affecting the liver, spleen, bone marrow, and central nervous system [2].

Pulmonary involvement in APH is exceedingly uncommon and may closely mimic a variety of inflammatory, cystic, or neoplastic processes, creating a significant diagnostic challenge [3]. Recent case reports have further broadened the pulmonary spectrum by describing solitary pulmonary nodules harboring EML4: ALK fusion and cases associated with concurrent cystic lung disease in the setting of Birt-Hogg-Dubé syndrome, underscoring the morphologic and clinical heterogeneity of thoracic presentations [4,5]. While identification of ALK rearrangements has important therapeutic implications and often guides targeted treatment with tyrosine kinase inhibitors (TKIs), such as crizotinib or alectinib, particularly in disseminated disease, the optimal management strategy for isolated, localized visceral involvement remains less well defined [3,6].

Here, we report a rare case of extrapulmonary APH managed with surgical resection. This case reports a rare localized thoracic presentation of APH and underscores the importance of comprehensive histopathological and molecular evaluation in atypical lesions, and highlights the potential role of surgery as a definitive treatment modality in selected localized disease.

## Case presentation

This is a 53-year-old female patient, known to have diabetes and a history of appendectomy, who presented to the thoracic surgery clinic complaining of pleuritic chest pain and mild shortness of breath on exertion; there were no other respiratory symptoms. She was vitally stable, and upon examination, the only finding was decreased air entry at the right base. Imaging was done, and the CT scan showed a massive heterogeneous tumor paravertebral in the lower right lung with sub-occlusion of the main right bronchus, and no metastasis above or under the diaphragm (Figure 1). A biopsy of the lesion showed a spindle cell lesion with histiocytic differentiation; the tissue showed relatively bland spindle cell proliferation with patchy foamy cytoplasm arranged in nests and scattered inflammatory cells (lymphocytes, plasma cells, and mast cells). A large immunohistochemical panel was performed and showed an ALK1 weak-to-moderate dot-like predominantly cytoplasmic expression. In addition, the lesional cells were positive for CD68, D2-40, Factor 13, and CD163, while being negative for the other significant markers.

**Figure 1:**
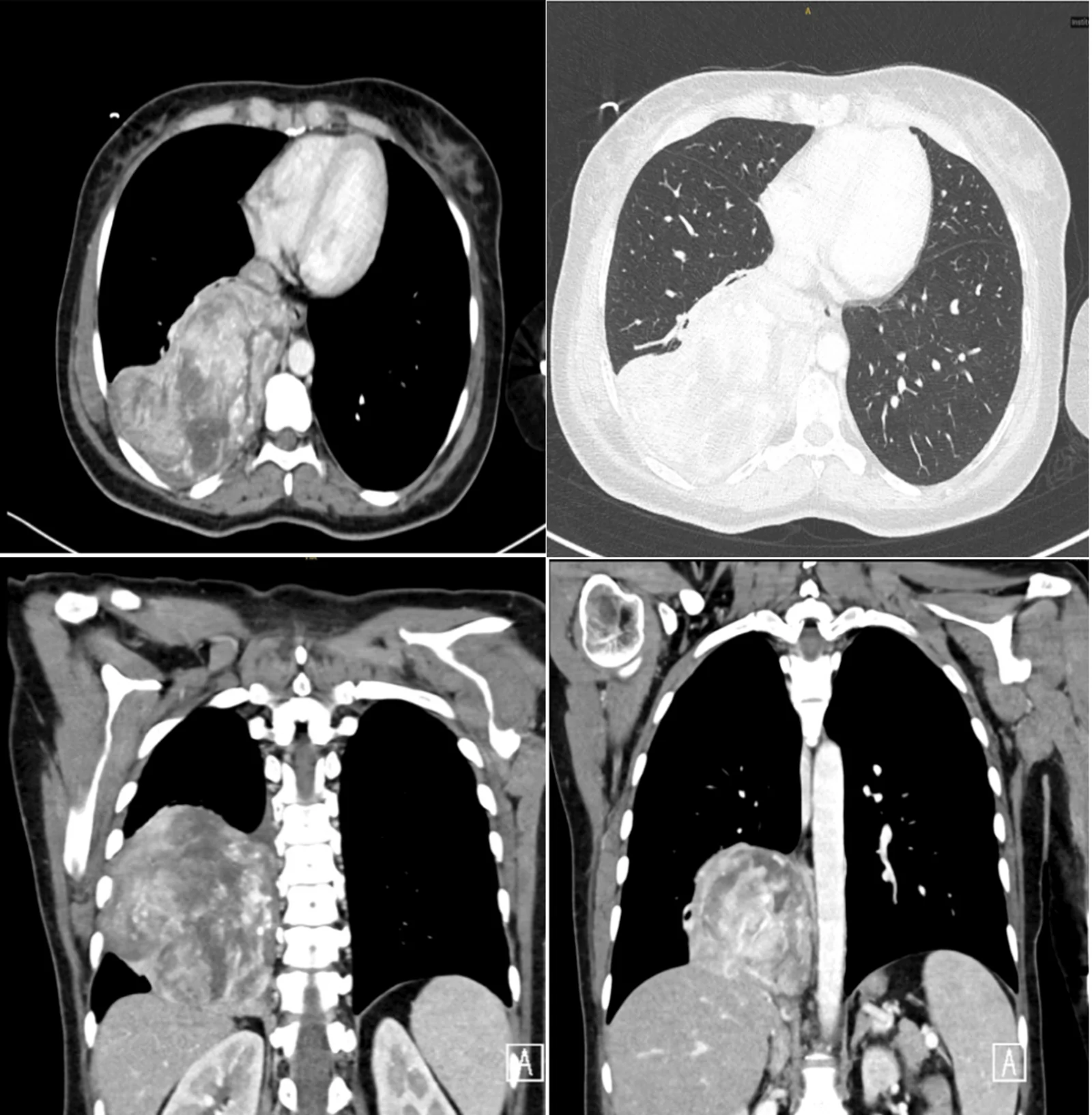
Massive heterogenous tumor paravertebral in the lower right lung with sub occlusion of the main right bronchus

A bone marrow biopsy was performed, and the result showed a normocellular marrow with active, maturing trilineage hematopoiesis and no definitive evidence of increased blasts, morphologic dysplasia, or abnormal infiltrates. Also, Immunostains such as CD20 and CD3 highlight scattered B-cells and T-cells, respectively. On the other hand, CD68, Factor 13a, and ALK showed no atypical histiocytic proliferation.

A PET CT was done and showed a markedly intense, metabolically active right pleural mass measuring 9.4 x 11.6 x 12.4 cm, with small right pleural effusion, along with interval enlargement as compared to the previous CT scan. Additionally, PET CT showed no evidence of nodal or distant metastases (Figures 2, 3).

**Figure 2:**
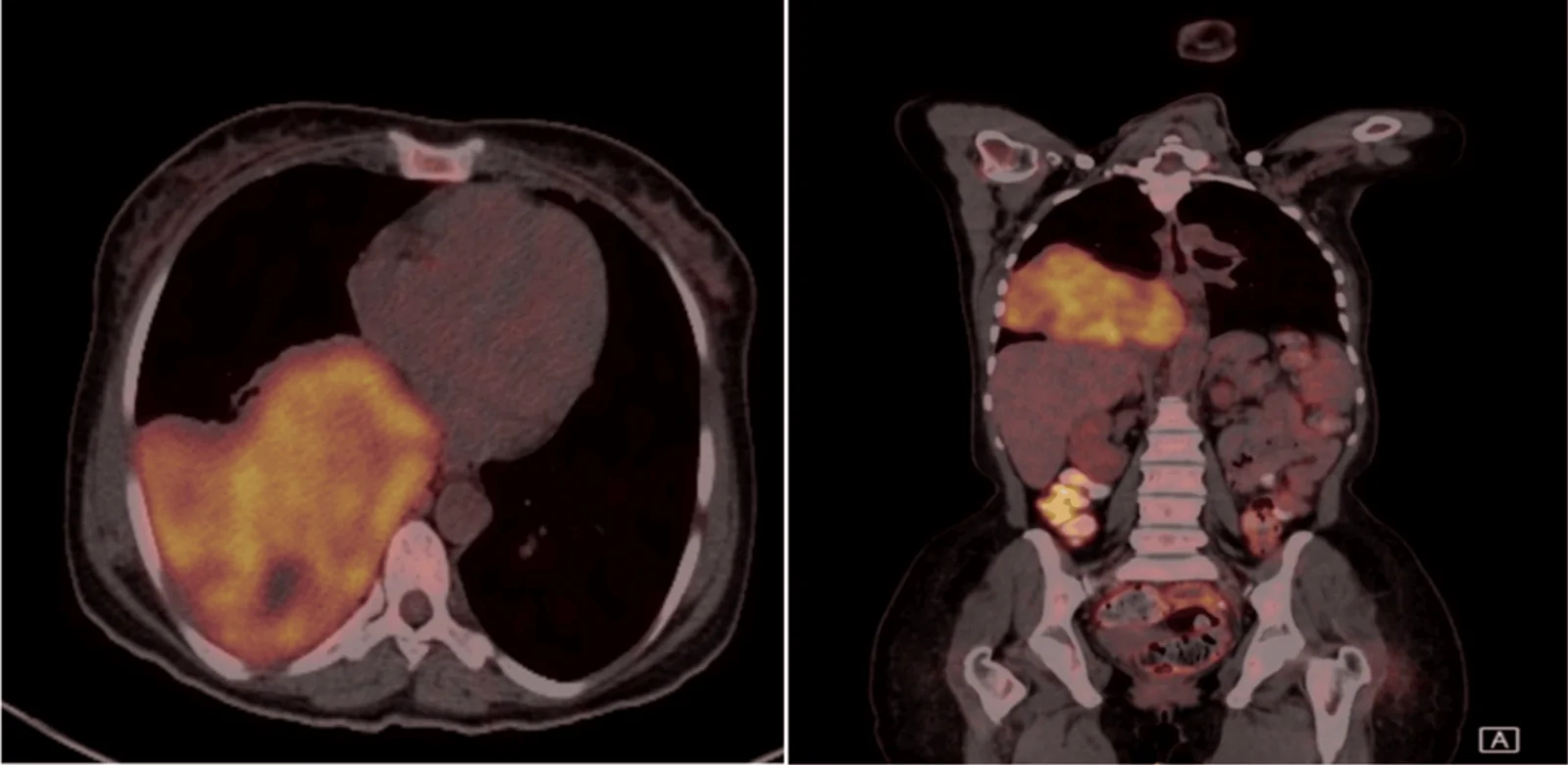
Markedly intense metabolically active right pleural mass

**Figure 3:**
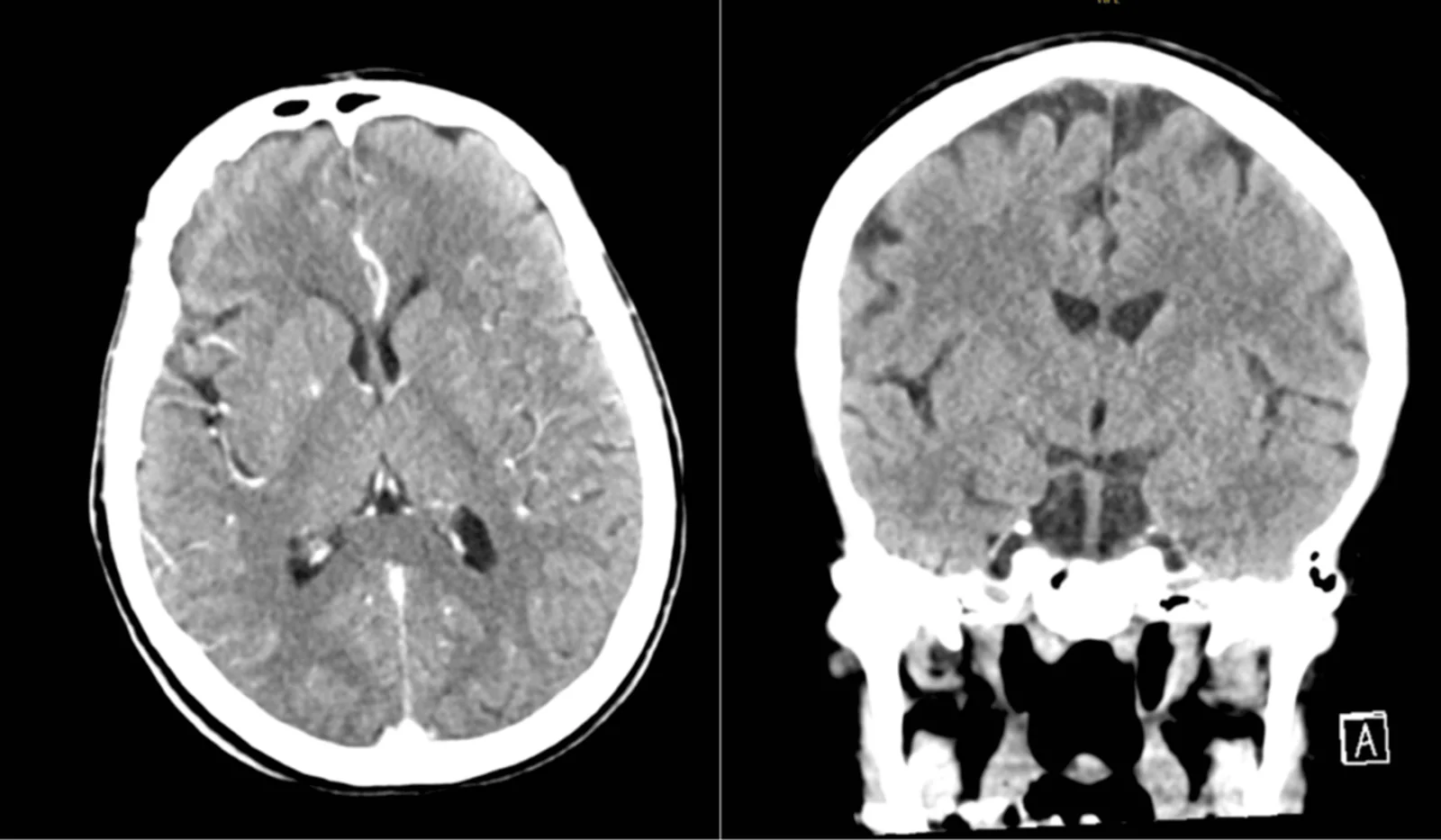
CT brain showed no evidence of intracranial metastasis

Finally, a thoracic spine MRI was done to complete the workup and make sure there is no intravertebral invasion of the tumor, and it showed no intraspinal extension or vertebral erosions noted (Figure 4). The case was discussed with the hematology tumor board, and due to the rarity of this case and after a thorough review of the literature, it was decided that the best approach is to proceed with a surgical resection of the lesion.

**Figure 4:**
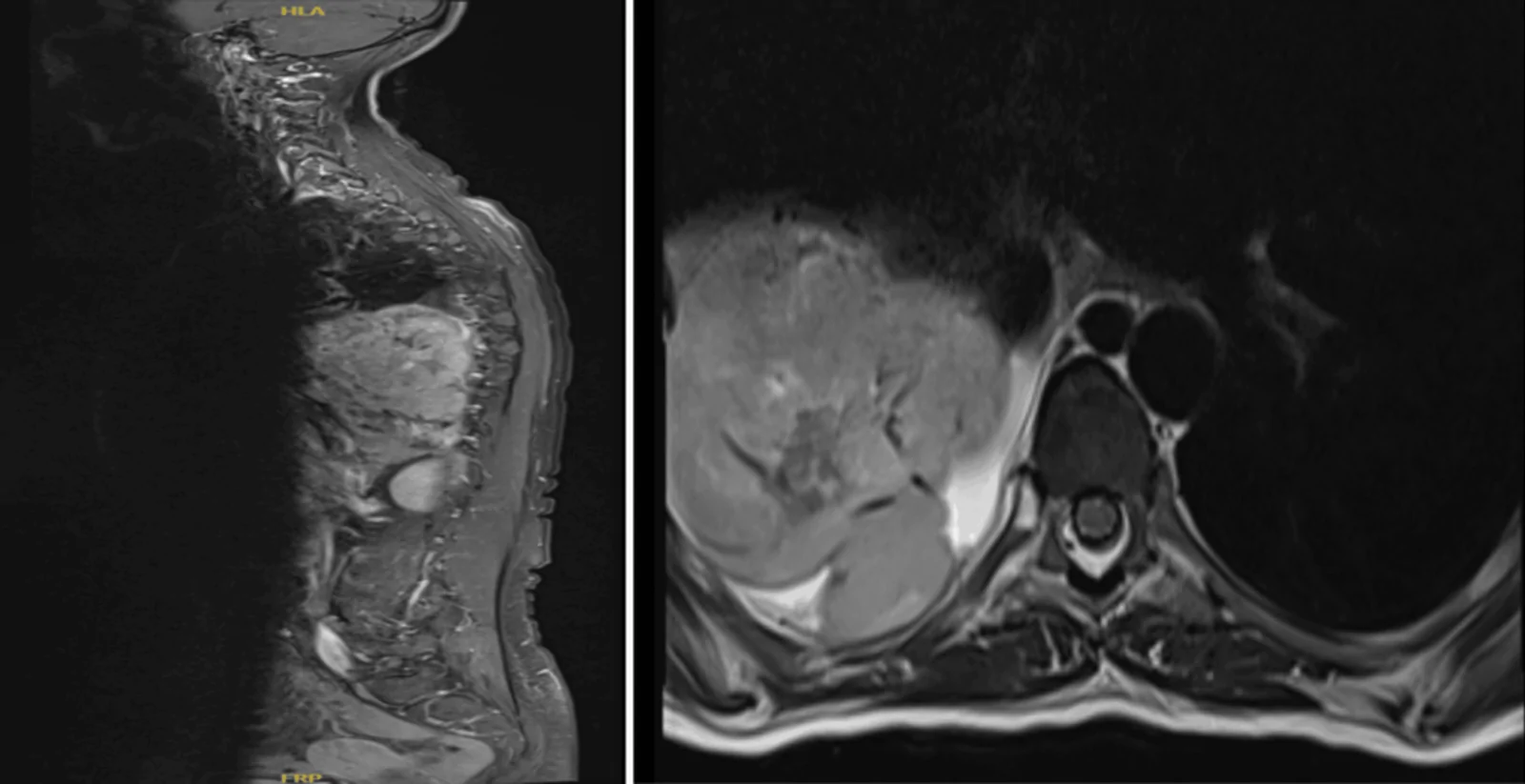
MRI of the spine showed no intraspinal extension or vertebral erosions noted

The operation commenced with the insertion of a thoracic epidural catheter for perioperative analgesia. Anesthesia was administered through a left-sided double-lumen endotracheal tube inserted for the purpose of securing one-lung ventilation during pulmonary surgery. Full monitoring capacity was instituted with continuous ECG, blood pressure, and transcutaneous oxygen saturation measurements.

Under strict aseptic precautions, the patient was positioned in the left lateral decubitus position with appropriate padding of all pressure points. The right hemithorax was prepped and draped in the usual sterile fashion. A right posterolateral thoracotomy incision was made. A serratus anterior-sparing technique was employed, with careful mobilization and retraction of the muscle fibers rather than division. Entry into the thoracic cavity was achieved through the sixth intercostal space. The intercostal muscles were divided using electrocautery, with meticulous care taken to preserve the neurovascular bundle along the inferior rib margin. A rib spreader was inserted and gradually opened to optimize visualization while minimizing undue stress on the ribs.

Upon entering the pleural cavity, a large, well-encapsulated, rounded mass was immediately visualized occupying a significant portion of the lower hemithorax. Exploration of the thoracic cavity was systematically performed, during which an additional pleural-based lesion was identified that had not been detected on preoperative imaging.

Given the unexpected intraoperative finding, a hematology consultation was obtained. Following multidisciplinary intraoperative discussion, the decision was made to proceed with complete surgical resection of both lesions, with a plan for possible adjuvant targeted therapy postoperatively.

Attention was first directed toward the larger mass. Dissection was initiated by incising the overlying pleura and developing a plane between the mass capsule and surrounding structures. A combination of blunt dissection using sponge sticks and a suction tip and sharp dissection with scissors and electrocautery was utilized to carefully mobilize the mass circumferentially. Particular attention was paid to the diaphragmatic surface, where dense fibrous adhesions were encountered. These were meticulously lysed using electrocautery and sharp dissection to avoid diaphragmatic injury.

The most significant attachment was identified between the mass and the right lower lobe parenchyma. At this stage, the possibility of a formal right lower lobectomy was considered due to the extent of adherence. However, careful dissection allowed delineation of a plane between the majority of the mass and the lung tissue. Residual parenchymal involvement was limited to a small area, which was addressed using an Endo-GIA (Medtronic, Minneapolis, MN, USA) stapling device. A wedge resection of the involved segment of the right lower lobe was performed. Multiple firings of purple-load Endo-GIA staplers were required to safely divide the thickened lung parenchyma. Each staple line was inspected for integrity and hemostasis. The mass was noted to be highly vascular, and intermittent bleeding from small vessels was encountered during dissection; this was effectively controlled using a combination of electrocautery, LigaClips (Teleflex, Wayne, PA, USA), and direct pressure. Following complete mobilization, the mass was delivered and removed from the thoracic cavity. The operative field was irrigated, and careful inspection confirmed adequate hemostasis. Attention was then turned to the pleural-based lesion. The lesion, along with a margin of surrounding parietal pleura, was excised using sharp dissection and electrocautery, ensuring complete removal. No invasion into deeper structures was noted, and the specimen was removed intact (Figure 5).

**Figure 5:**
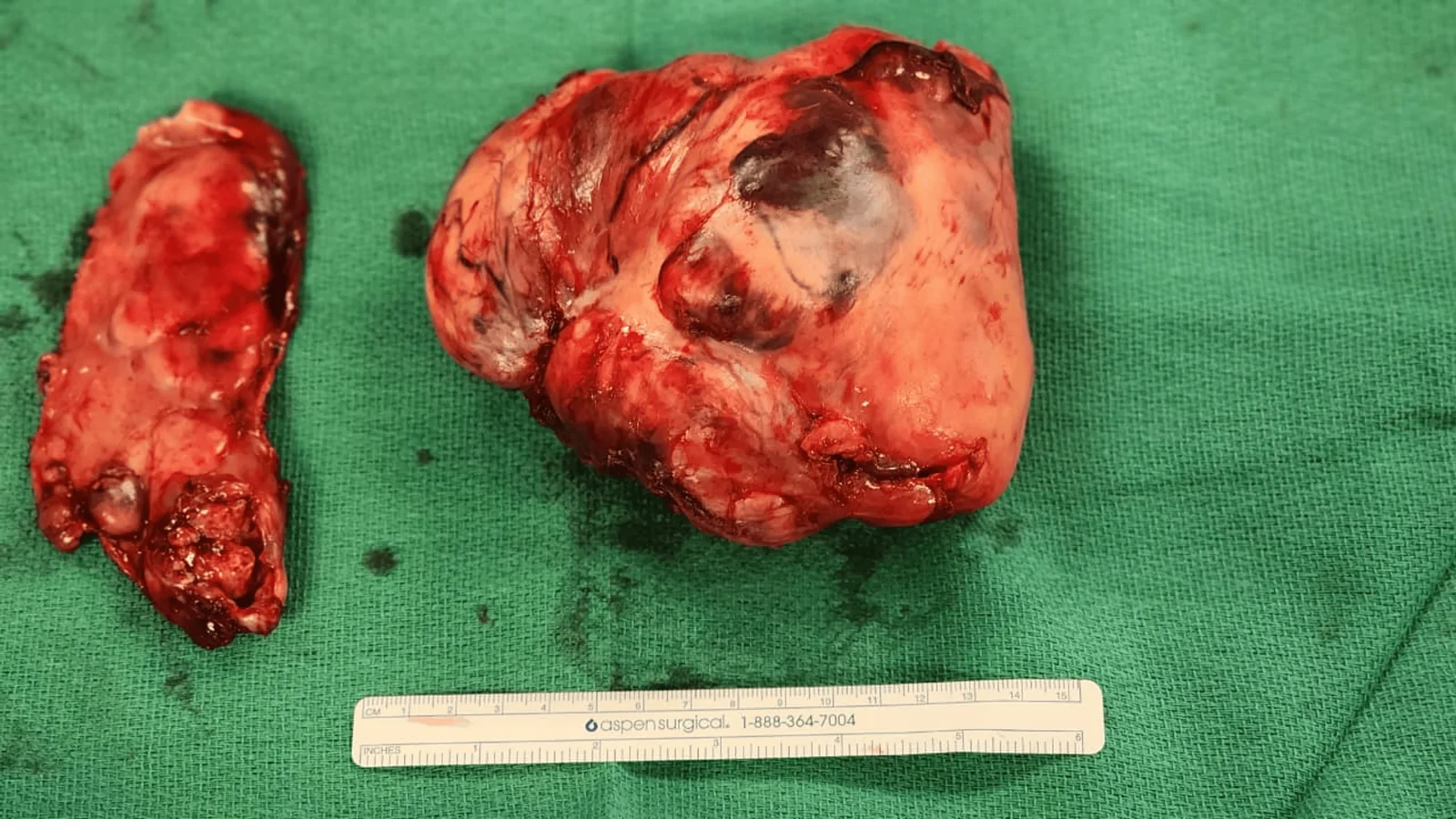
Right lower lobe mass and pleural based lesion

A thorough exploration of the thoracic cavity was subsequently performed, including inspection of the lung surfaces, diaphragm, mediastinum, and pleural reflections, with no gross residual disease identified. The staple line on the lung was submerged under saline and subjected to positive-pressure ventilation to assess for air leak; none was observed. Hemostasis was reconfirmed throughout the operative field. A 28-French intercostal chest tube was inserted through a separate stab incision in the inferior thoracic wall and directed posteriorly and apically.

The thoracotomy incision was closed in layers, and the patient tolerated the procedure well, with stable intraoperative parameters.

Post-operatively, the patient was doing very well, with pain being controlled and a satisfactory chest X-ray. The chest tube was removed on post-operative day two, and the patient was discharged home in excellent general condition.

Pathology review showed that all specimens’ margins were free of tumor. Also, the tumor appeared to be arising from the extrapulmonary space with invasion of visceral pleura and lung parenchyma. At first the main differential diagnosis for this lesion was inflammatory myofibroblastic tumor (IMT), however, immunohistochemical stains were performed and show that the tumor cells were positive for ALK1, ALK (D5F3), CD163, CD68, and Factor 13, CD4, and S100 (patchy), while being negative for SMA, MSA, desmin, myogenin, MyoD1, calponin, EMA, CD34, CKpan, and CAM5.2, making IMT unlikely to be the diagnosis. Then, fluorescence in situ hybridization (FISH) testing was performed on the prior specimens, and the results showed ALK rearrangement. As a result, in combination with morphologic and immunohistochemical findings, APH was the diagnosis.

The patient followed up in both the thoracic and hematology clinics regularly afterward and was doing very well. A repeated PET scan was done two months after the surgery, showing no evidence of residual or recurrent disease (Figure 6). The case was discussed among colleagues in hematology, and in the absence of any signs of recurrence, there was no need to give any adjuvant treatment; the plan was to continue surveillance in the form of PET/CT every six months for two years and then yearly thereafter.

**Figure 6:**
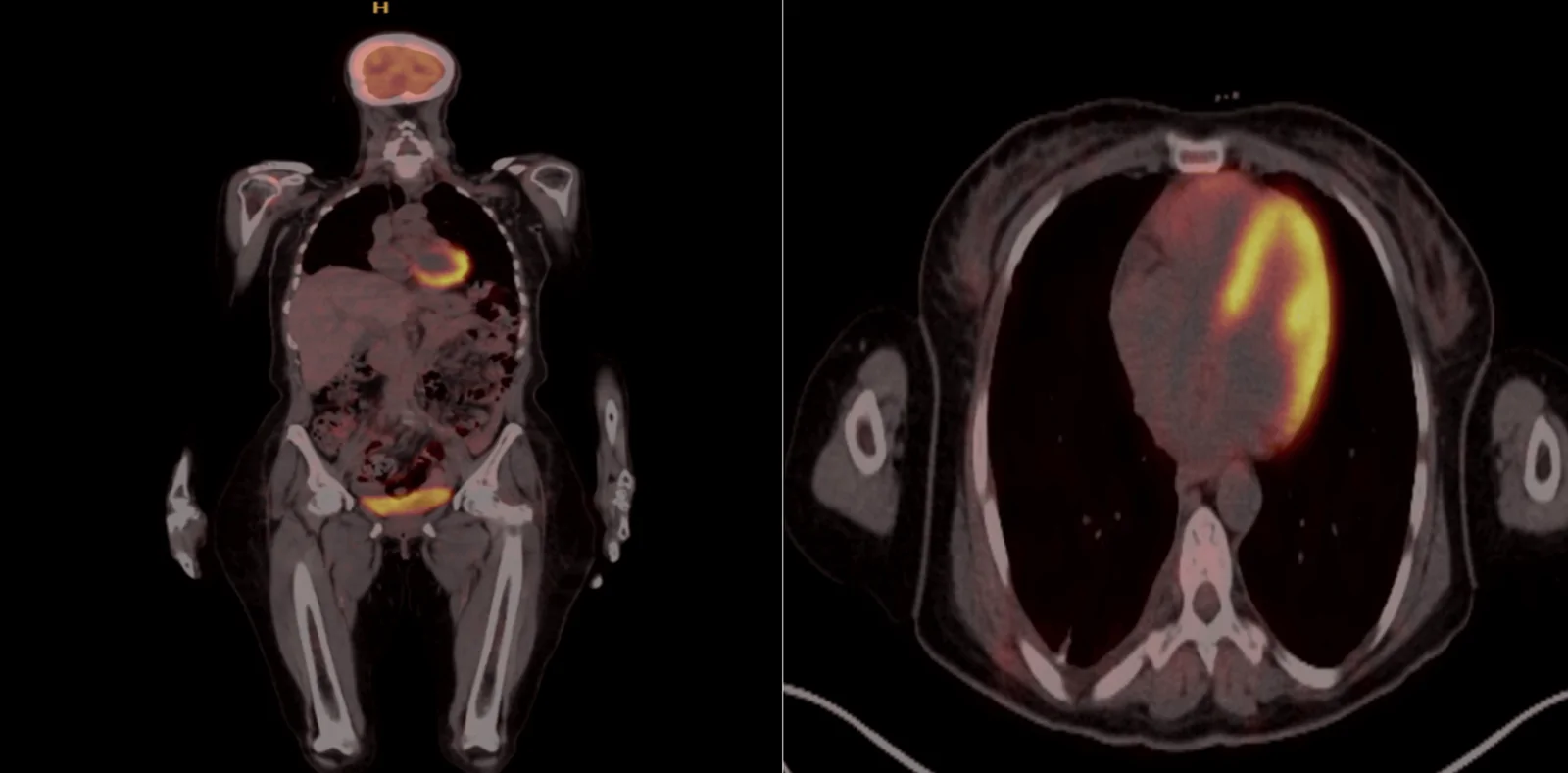
PET-CT two months after the operation showed no evidence of residual or recurrent disease

## Discussion

Histiocytoses are rare disorders defined by the accumulation of cells believed to be generated from dendritic cells (DCs) or macrophages. Their clinical behavior can range from mild to widespread, and in certain cases, life-threatening. There are three categories: Langerhans cell (LC), non-LC-related, and malignant histiocytoses (MH). APH is a rare subtype that was previously misclassified as juvenile xanthogranuloma (JXG) or other histiocytic disorders, characterized by translocations involving the ALK gene, most commonly KIF5B::ALK, which leads to constitutive activation and uncontrolled proliferation [1]. Since such cases take part in the MAPK pathway activation, the histological presentation is vast and diverse with slight differentiation across organs. For example, the majority of cases exhibit the typical features of xanthogranuloma; microscopy reveals foamy macrophages, large Touton cells, and, occasionally, cells resembling epithelial cells. The oval-shaped nuclei of the foamy cells are eosinophilic in nature and may exhibit modest nuclear membrane folding or indentation; mitotic features are uncommon. Additionally, some spindle cells with ovoid or elongated nuclei, eosinophilic cytoplasm, and hazy boundaries are visible. In our case, biopsy showed a spindle cell lesion with histiocytic differentiation, sporadic inflammatory cells (lymphocytes, plasma, and mast cells), and relatively bland spindle cell growth with patchy foamy cytoplasm organized in nests [7].

According to recent studies, it is reported that fewer than 100 cases have been confirmed, with the majority being females [7]. The clinical presentation of the disease depends very much on the age of the patient. In infancy, most cases have a multisystem disease with hepatomegaly, anemia, and thrombocytopenia being the hallmark of presentation. Some may present with other manifestations, such as splenomegaly, leukocytosis, skin lesions, and lung involvement seen as interstitial lung disease. In adult cases, visceral disease of the lung, bone, and central nervous system is commonly reported [8]. Some patients experienced complete remission after receiving second-line systemic treatment that included anabolic acid, immunosuppressants, and steroids. Others achieved spontaneous regression with supportive care, such as blood transfusions alone. The remaining patients exhibited variable degrees of improvement after receiving treatment regimens like chemotherapy; however, because too few patients are known, the efficiency of the various therapies is still being investigated [5]. In children and adults, APH can be seen in the nervous system, bone, lung, liver, skin, and lymph nodes, with the usual presentation being dissemination affecting one or multiple systems. Conventional systemic therapy alone, or in conjunction with an ALK inhibitor, is the first-line treatment for patients with multisystem involvement. In addition to standard systemic therapy, surgical excision is required for patients with unisystemic involvement [9]. Thoracic APH is extremely rare and may present without typical symptoms, so it may be identified only through imaging [10]. In our study, we report a case of APH presenting as a pleural-based mass. The patient presented to the hospital complaining of pleuritic chest pain and mild shortness of breath on exertion, with decreased air entry at the right base; other systems were intact, and since imaging showed a single pleural mass with no evidence of nodal or distant metastases, we determined that a surgical resection of this lesion would be the best course of action for her.

It is crucial to have a precise diagnostic criterion to differentiate APH from a number of other diseases, because of its various clinical manifestations and the variety of cell types that vary in different disease sites. The clinical history, along with morphological and immunohistochemical characteristics, is used to make the diagnosis (Figure 7).

**Figure 7:**
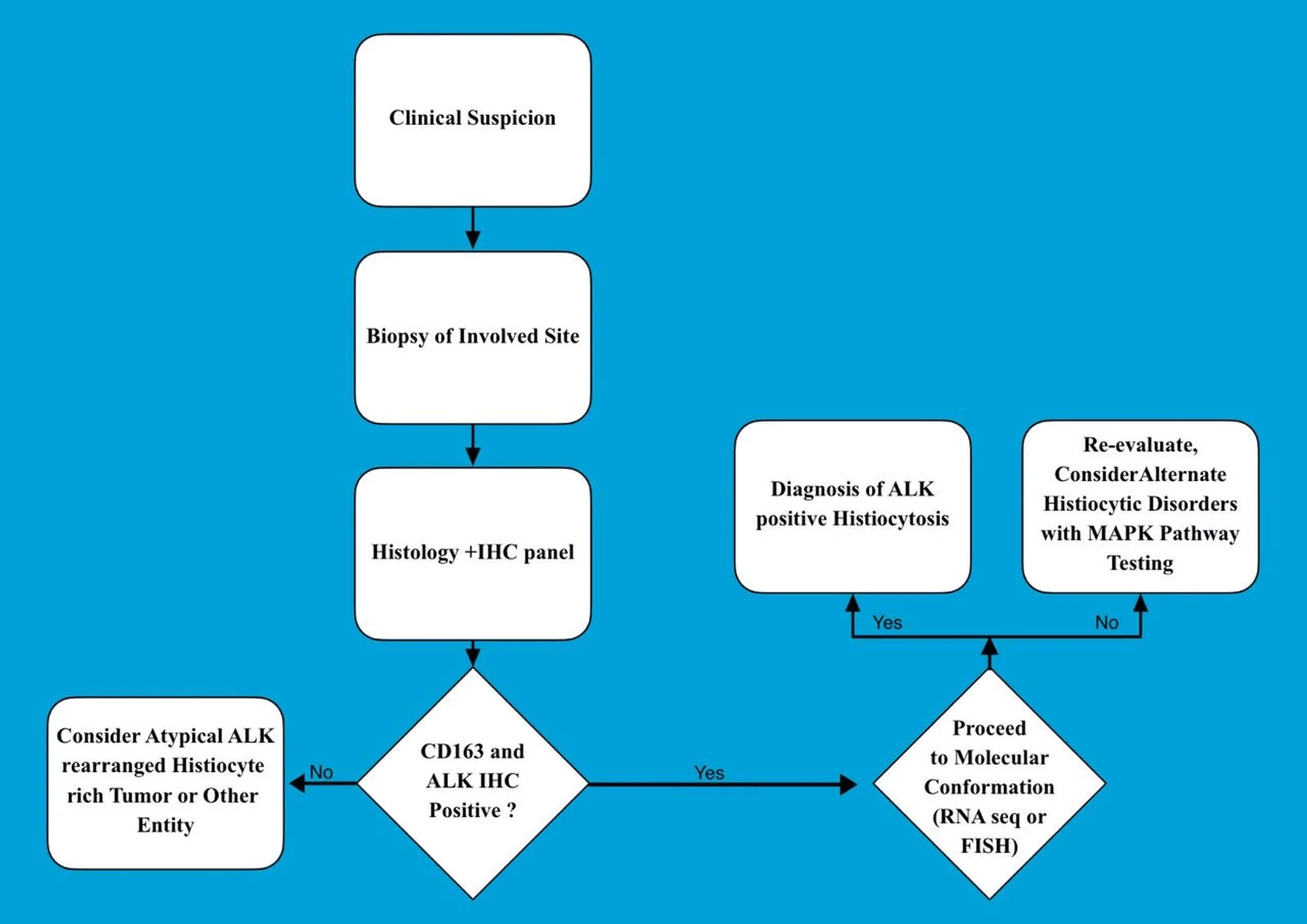
Diagnosis summary FISH: fluorescence in situ hybridization; IHC: immunohistochemistry

First, the clinical suspicion of APH depends on the clinical manifestations seen in patients as discussed above. Second, biopsy, preferably excisional or core biopsy, from the involved site (skin, soft tissue, bone, lymph node, etc.) for morphological and immunohistochemical assessment. Regarding the histological patterns, a classic xanthogranuloma-like pattern, seen as plump foamy histiocytes, Touton giant cells are common, as well as sheets of histiocytes with ovoid nuclei showing “cup-shaped” indentations, minimal atypia, low-to-moderate mitotic activity, and no lipidization. Spindled or epithelioid morphology may also occur. Furthermore, an immunohistochemical panel is essential for diagnosis. CD163 defines the histiocytic lineage, making it impossible to diagnose without it; other markers support the diagnosis, such as CD14 and CD4, but they need additional macrophage markers. In addition, some markers, like p-ERK (phospho-ERK), Cyclin D1, S100, OCT-2, etc., may be misleading. Because of that, ALK IHC alone is insufficient for diagnosis; cases with ALK staining but no rearrangement may represent other histiocytoses, which will affect treatment. Third, molecular confirmation is necessary for definitive diagnosis and to direct targeted therapy since ALK IHC can be positive in the absence of an ALK rearrangement (and vice versa). Because it can detect all partners and identify both known and new fusions, RNA-based next-generation sequencing (RNA-seq) is the gold standard, but it requires high-quality RNA; tissue is desirable if it is available. Although DNA-based sequencing can detect certain fusions, RNA sequencing is more sensitive in this regard. FISH can be used, and it is widely available, but it does not identify the fusion partner and may miss cryptic rearrangements. Lastly, some tumors have the ALK rearrangement pattern, but lack the expression of the markers on the ALK cells; instead, the markers are intermixed [11]. These cases should not be classified as APH, since they may represent other entities (Table 1). In addition to that, APH may mimic the presentation of different diagnoses, including Erdheim-Chester disease (ECD). In a published study in the literature done by Syrykh et al., a case of APH associated with CLL/SLL showed an ECD-like presentation and remained disease-free after four years of ibrutinib therapy, a Bruton Tyrosine Kinase (BTK) inhibitor, which could be effective for the treatment of APH [12].

**Table 1:** APH differential diagnosis APH: ALK-positive histiocytosis

Disease	Distinguishing features
Erdheim-Chester disease	Older adults (median 55 y), male predominance, periaortic infiltration, BRAF V600E common; ALK rearrangements absent.
Langerhans cell histiocytosis	CD1a⁺, langerin⁺, Birbeck granules; BRAF/MAP2K1 mutations; ALK negative.
Juvenile xanthogranuloma (systemic)	Usually, young children; ALK rearrangements may occur but are less frequent; histology similar but without ALK fusion often shows other MAPK mutations.
Rosai-Dorfman disease emperipolesis	S100⁺, OCT-2⁺, but ALK negative; KRAS/MAP2K1 mutations.
Histiocytic sarcoma	Malignant cytology, high mitotic rate; can harbor ALK rearrangements rarely, but morphology and clinical behavior differ.
Epithelioid fibrous histiocytoma	ALK-rearranged (often EML4-ALK), but confined to the dermis, lacks CD163 expression.

APH has no standardized treatment guidelines. Current practice includes surgical resection, ALK inhibitors, and chemotherapy, and the management plan mostly depends on the clinical presentation and disease severity as mentioned above. Prognosis differs widely and is generally favorable, especially in a single-system disease [5]. Most cases reported in the literature with a single organ involvement, easily resectable and accessible lesions, underwent surgical excision with a complete response and no evidence of recurrence or progression. However, multisystem cases were more often treated with chemotherapy and ALK inhibitors, with outcomes ranging from partial response to disease progression (Table 2) [5,8,9,12-21].

**Table 2:** Literature review of the most relevant articles

Reference	Case no.	Age/ gender	Site of disease	Diameter	Therapy (primary)	Further treatment	Follow up	Outcome
Yang et al. (2025) [5]	1	61y F	Lung (right lower lobe)	2.5 cm	Surgical resection	No further treatment	8 months	No evidence of recurrence or metastasis
Zhou et al. (2023) [8]	2	46y F	Breasts, lungs, brain, and bones	Breasts (measuring 1-2 cm), lung (largest measuring 0.9 cm)	Crizotinib	No further treatment	Nearly 2y	Complete response
Tian et al. (2021) [13]	3	51y F	Lung, brain, and lymph nodes	Brain (1.3 x 1.7 x 1.5 cm), lung (largest measuring 1 cm)	Surgical resection	Alectinib	10 months	No evidence of progression and patient had durable remission
Chang et al. (2019) [3]	4	40y F	Breast	Not clearly reported	Surgical resection	Not reported	3.5y	Complete response
Gupta et al. (2019) [15]	5	50y F	Appendix	1.5 x 1.5 cm	Surgical resection	Not reported	Limited follow up (NS)	Complete response
Jaber et al. (2021) [16]	6	27y M	Intradural extra-medullary, nerve root (L3)	1.5 cm	Surgical resection	Not reported	9 months	Complete response
Syrykh et al. (2021) [12]	7	37y F	Breast and bone (ECD-like)	Not clearly reported	CLL treatment	Ibrutinib	4y	Complete response
Qui et al. (2021) [17]	8	49y M	CNS, bone, soft tissue, visceral organs, pleura	Not clearly reported	γ-knife, chemotherapy, ALK inhibitor	Not reported	Nearly 2 months	Clinical response
Tran et al. (2021) [18]	9	20y F	Mesentery	13 cm	Surgical resection	Not reported	1y	Complete response
Bai et al. (2021) [14]	10	52y F	Lung	1.3 cm	Surgical resection	Patient refused any further treatment	5 months	Complete response
Zhu et al. (2021) [19]	11	18y F	Umbilicus	11.7 mm x 8.3 mm x 11.8 mm	Surgical resection	Not reported	18 months	Complete response
Li Y et al. (2022) [20]	12	32y M	Lung, liver, pancreas, skin, gallbladder, kidney	Not clearly reported	Alectinib	Not reported	27 months	Partial response
Kemps et al. (2022) [9]	13	19y F	CNS/PNS, bone, lung, liver, lymph node, breast, pancreas	Not clearly reported	Chemotherapy (2-CDA, 3 cycles) then ALK inhibitor (Alectinib)	Not reported	2y	Partial response
	14	21y M	Liver, skin, colorectal involvement	Not clearly reported	High dose corticosteroids	Not reported	2 months	Partial response then died of sepsis
	15	28y F	CNS/PNS, bone	Not clearly reported	Alectinib	Not reported	9 months	Partial response
	16	29y M	PNS, bone	Not clearly reported	Corticosteroids followed by pegylated interferon-a	Brigatinib	2.5y	Complete response
	17	20y M	CNS (right frontal lobe)	Not clearly reported	Surgical resection	Not reported	10 months	Complete response
	18	41y F	Bone	Total size more than 10 cm	Radiotherapy	Crizotinib then chemotherapy (VBL/PRED) then Alectinib	2y	Partial response
Liu et al. (2023) [21]	19	38y M	Lung, mediastinum, lymph nodes	Lung (5.5 x 5 cm)	Chemotherapy	Alectinib and lorlatinib	Lost follow up	Stable for 24 months then progressive disease for 2 months
Current study	20	53y F	extrapulmonary	Right pleural mass measuring 9.4 x 11.6 x 12.4 cm	Surgical resection	No further treatment needed	2 months	No evidence of residual or recurrent disease

APH is characterized by ALK rearrangements leading to constitutive activation and uncontrolled proliferation of histiocytic cells; thus, ALK inhibitors are an appropriate choice. Crizotinib is a first-generation FDA-approved tyrosine kinase inhibitor that targets ALK and is generally well-tolerated by patients. In the literature, Zhou et al. reported a case of a 46-year-old female patient who was diagnosed with APH with the presence of the disease in her breasts, lungs, brain, and bones. She was started on Crizotinib and was followed up with a complete response and no further evidence of disease [8]. In another study done by Kemps et al., a 41-year-old female patient with bone involvement was started on radiotherapy as a primary treatment, then went to Crizotinib as a secondary therapy [9]. Unfortunately, the patient developed a severe anaphylactic shock that required resuscitation on day one of Crizotinib administration. Then, the patient received four courses of vinblastine/prednisone-based chemotherapy with progressive disease, and after that switched to alectinib as an ongoing therapy and was on disease regression for two years of follow-up [9]. In the case of APH with disseminated disease, Tian et al. reported a case that responded well to alectinib [13]. The study documented a female patient who had APH with lung, intracranial, and lymph node involvement. The patient underwent surgery for the left frontal tumor, then received treatment with a second-generation ALK inhibitor (alectinib) and showed no evidence of disease progression and had actually durable remission [13].

In our study, we reported a rare localized thoracic APH and illustrate that surgical intervention is reasonable in selected APH cases with localized disease. Our patient had a pleural mass with no evidence of metastases, so as mentioned above, surgical resection of the lesion was the best approach for her case. In addition, there was no recurrence, and the patient had a complete response, so there was no need to start her on any adjuvant treatment, and surveillance is the best option for her in the long term. A study done by Yang et al. documented a similar case of pulmonary APH who presented to the hospital ill, with a chest CT scan that revealed multiple bullae in both lungs, with the presence of pulmonary nodules in the right lower lobe [5]. The patient was initially diagnosed with an inflammatory myofibroblastic tumor (IMT) and underwent right lower lobe wedge resection, and the resected nodule was taken for further investigation. Postoperatively, FISH and second-generation sequencing confirmed the diagnosis of APH combined with Birt-Hogg-Dubé syndrome (BHD). The patient did not receive any further treatment and was disease-free for the follow-up period [5]. In another study reported by Bai et al., a case of localized APH was discovered accidentally in a Chinese female patient, for which she underwent surgical resection [14]. After surgery, the patient refused any other treatment, was followed up for nearly five months, and showed no surgical complications or disease recurrence [14].

## Conclusions

In conclusion, in this case report, a complete surgical resection was performed effectively for the patient, and this may be considered in some localized, resectable, thoracic ALK-positive histiocytosis (APH) cases after multidisciplinary investigations and molecular testing confirmation. Thus, this study emphasizes the importance of early comprehensive histopathological and molecular studies to confirm the diagnosis of APH and the role of surgical resection as a potential management approach in selected localized cases. However, our literature review was limited to adult-relevant APH cases, as the pediatric population was not included. Also, we excluded non-English-language articles. Further research is warranted to collect more cases, evaluate management strategies, and assess patients' prognosis.
